# Which effects had the pandemic in migrants’ health and well-being? A mixed-methods approach

**DOI:** 10.1093/eurpub/ckac129.606

**Published:** 2022-10-25

**Authors:** A Gama, MJ Marques, AR Pedro, LV Hoffmeister, F Rodrigues, JS Ribeiro, S Dias

**Affiliations:** 1 NOVA National School of Public Health, PHRC, Universidade NOVA de Lisboa, Lisbon, Portugal; 2 Comprehensive Health Research Center, Lisbon, Portugal; 3 NOVA National School of Public Health, Universidade NOVA de Lisboa, Lisbon, Portugal

## Abstract

**Background:**

The COVID-19 pandemic affected populations’ health, with a disproportionate impact on those most socially vulnerable such as migrants. The way these populations experienced the pandemic lockdowns and its effects on daily life are yet to be known. This study aimed to understand the effects of the pandemic on health and well-being of migrants in Portugal.

**Methods:**

In a mixed-methods approach, a survey was conducted with a community-based sample of 1126 migrants in the Lisbon Metropolitan Area, assessing sociodemographics, migration-related characteristics and the perceived impact of the pandemic on health. In addition, n = 12 migrants purposively recruited were invited to participate in a photovoice study, sharing photographs about their daily life during the lockdowns. Following semi-structured interviews were conducted. Quantitative data were analysed using multivariable analysis and qualitative data were analysed through content analysis.

**Results:**

A fifth of the participants perceived having worse health condition since the pandemic, which was more likely among women (OR = 1.58, CI95% 1.13-2.20), those >45 years old (OR = 1.78, CI95% 1.02-3.16), with lower education (Basic education: OR = 1.57, CI95% 1.01-2.47) and with lower monthly income (<EUR 650: OR = 1.69, CI95% 1.18-2.44). Two themes emerged from the photovoice: effects of the pandemic lockdowns on daily life (routines, social relations, work) and on health and well-being (eating habits, physical exercise, leisure). Strategies to cope with the adverse effects included social activation and changes in lifestyles.

**Conclusions:**

The pandemic had disproportionate effects on some migrant groups, intensifying social and health inequalities, with consequences for their well-being. Participatory methods can contribute to further understand migrants’ experiences while involving and empowering them for health promotion.

**Key messages:**

• The pandemic had adverse effects on migrants’ health and well-being, disproportionately affecting most socially vulnerable migrant groups.

• Participatory research methods as photovoice are valuable to gain access to individual experiences and perspectives, while involving and empowering participants.

